# Surgical Correction of Post-traumatic Residual Deformity of the Mandible: A Case Report

**DOI:** 10.7759/cureus.46710

**Published:** 2023-10-09

**Authors:** A Saneem Ahamed, Vijaya Lakshmi G, Nithin VM, Kapil Dev Kumar, Mubeena Syed

**Affiliations:** 1 Oral and Maxillofacial Surgery, Priyadarshini Dental College and Hospital, Chennai, IND; 2 Dentistry, Priyadarshini Dental College and Hospital, Chennai, IND

**Keywords:** open reduction, corrective osteotomy, callus, refracture, asymmetry, malocclusion, malunion, secondary deformities, post traumatic residual deformity, mandibular fracture

## Abstract

Mandibular fractures are the most common trauma cases that we often come across in our day-to-day practice of oral and maxillofacial surgery. Various factors can lead to deformities and make those cases more challenging, which includes a delay in surgical treatment, resulting in non-union or malunion of the fracture site causing occlusal disturbances and functional abnormalities in the temporomandibular joint.

## Introduction

A fracture is a wound that results from a bone breaking or cracking [1]. Despite being the largest and most robust facial bone​​​​[2], the mandible, due to its conspicuous anatomical location, plays a pivotal role in both masticatory function and facial aesthetics. Remarkably, it ranks as the third most commonly encountered maxillofacial fracture, contributing substantially to the spectrum of maxillofacial fractures, with prevalence ranging from 36% to 59% [3]. Various lower-face injuries cause mandibular fractures. These encompass accidental falls, especially in elderly patients; bike, scooter, or skateboard accidents during contact sports; jaw assault; and car crashes [1]. Mandibular fractures are classified based on distinct anatomical regions: the symphysis, body, angle, ramus, condylar process, coronoid process, and alveolar process [4]. Most people experienced a single fracture, mainly involving the parasymphysis (58%). Among those with multiple fractures (42%), the angle and parasymphysis combination (18%) was the most prevalent [3]. For the initial evaluation of mandibular trauma, panoramic radiographs provide a comprehensive mandibular view, including condyles, dentoalveolar complex, and dentition. They are crucial for detecting anterior mandibular issues frequently unnoticed in standard radiographic films. However, in cases of inadequate diagnosis with plain radiographs, CT scans and three-dimensional reformatted images may be considered [4]. Inflammation, chondrogenesis, osteogenesis, and remodeling are the four stages of the typical fracture healing process. During "bony union," proper realignment and fixation restore function and prevent deviations. Oral and maxillofacial surgery professionals are concerned that certain fractures might result in abnormal unions[5]. Facial fractures that are left untreated can lead to a variety of aesthetic and functional abnormalities [6]. The malunion of the fracture was caused by the delay in seeking medical attention and treatment [7]. Bone union in an incorrectly reduced position is referred to as malunion [8]. The most typical indication of malunion is malocclusion[8]. Age, gender, trauma type, fracture placement, and severity all have an impact on a patient's aberrant union [5]. Malunited mandibular fractures frequently result in malocclusion due to occlusal adaptation [9], yet correcting these deformities can enhance the patient's quality of life despite surgical hurdles [10]. Defects in the healing process, such as delayed union, malunion, or non-union, can result in residual abnormalities, emphasizing the need for surgical treatment. The surgical goals include restoring normal occlusion, attaining bone union, and correcting mandibular malposition. The primary objective of our case report is to meticulously assess the clinical presentation and management of a malunited parasymphysis and body of the mandible fracture, surgically assisted through open reduction and internal fixation (ORIF) while elucidating a straightforward treatment approach that can be readily applied in clinical practice.

## Case presentation

A 59-year-old male patient presented to the Department of Oral and Maxillofacial Surgery at Priyadarshini Dental College and Hospital with the primary concern of pain, swelling, and tooth mobility in the lower left posterior region. These issues had been ongoing for the past month, following a road traffic accident in which he sustained multiple injuries, including a leg fracture. Initial treatment for the leg fracture was provided at another hospital. The patient had a medical history of hypertension and was currently on medication for it. He was well-oriented and conscious.

**Figure 1:**
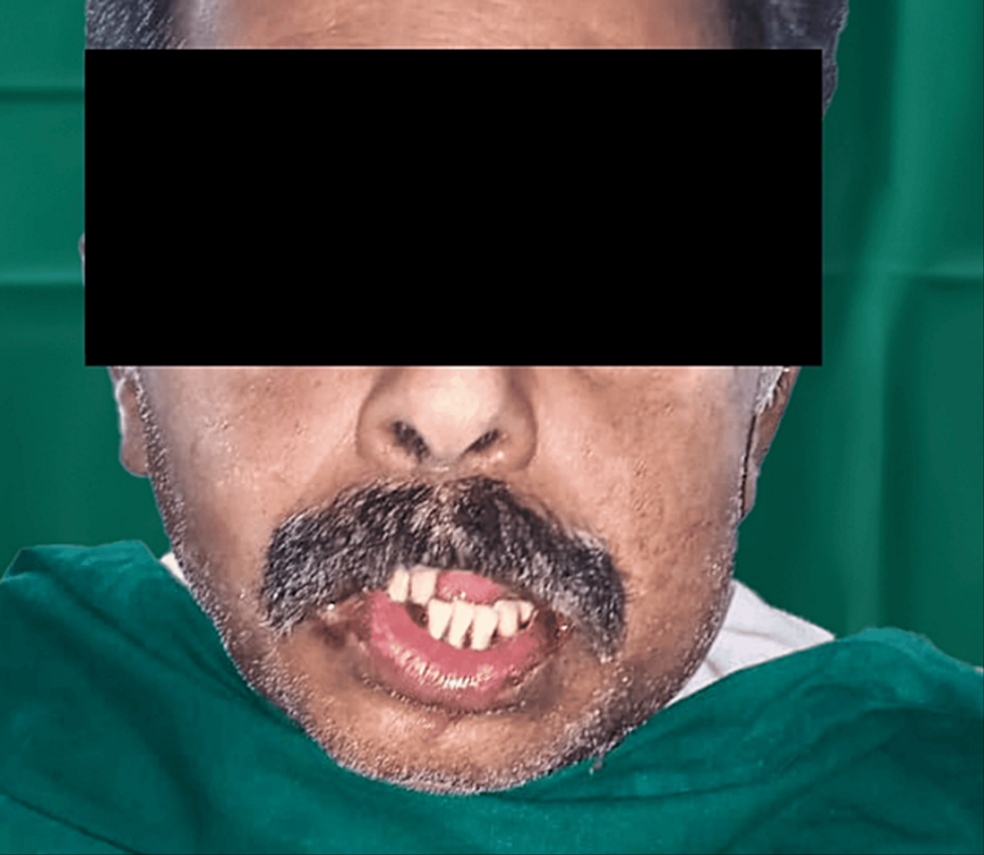
Preoperative photograph showing facial asymmetry (frontal view)

**Figure 2:**
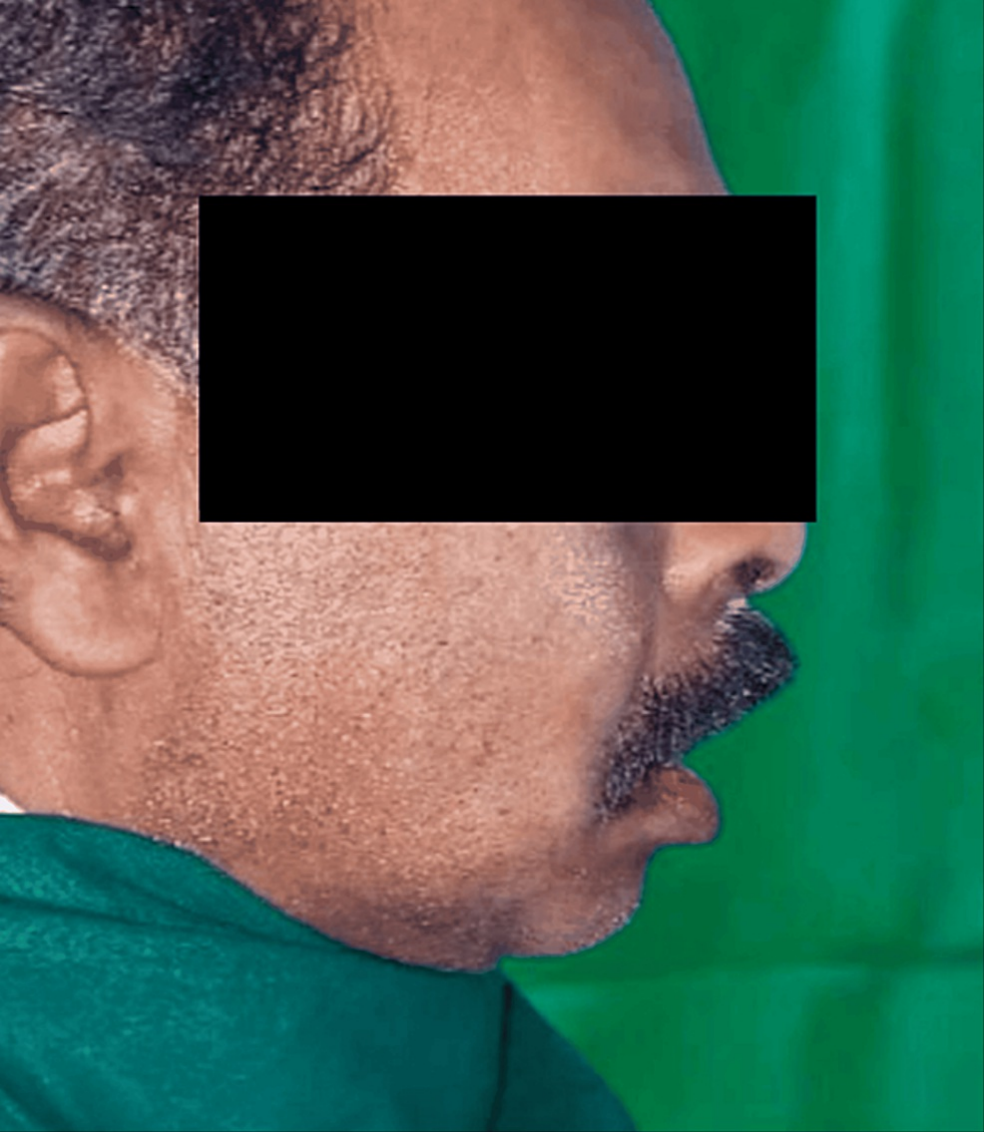
Preoperative photograph (lateral view)

Extraoral examination revealed facial asymmetry (Figures 1, 2).

Subsequent extraoral assessment confirmed the presence of a hard consistency swelling on the left side of the mandible upon palpation. The intraoral examination did not reveal any bleeding or pus discharge, but it did confirm the displacement of the mandible on the left side, along with the presence of an open bite on the left side. All findings were confirmed by palpation, revealing evident step deformity and the presence of tenderness. Significantly, there was no segmental mobility observed.

**Figure 3:**
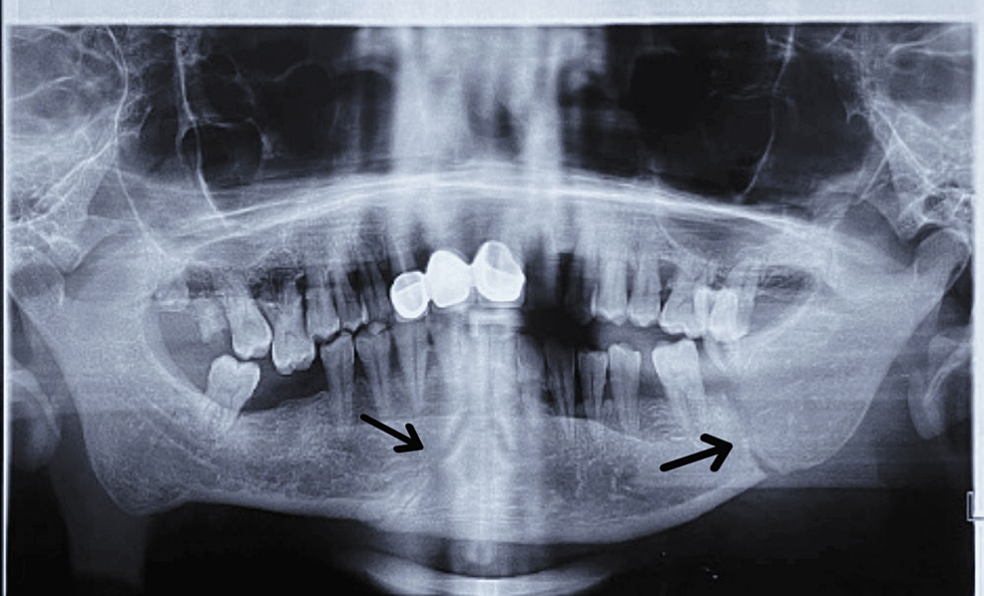
Preoperative Orthopantamograph showing radiolucent fracture lines with precision arrows.

A radiographic investigation was carried out to confirm the diagnosis. Orthopantomagram (OPG) was chosen for assessment due to its panoramic view, aiding in mandibular fracture assessment and approximate localization on radiographic analysis (Figure 3).

A radiolucent fracture line was seen on the left side body of the mandible in relation to 36 and 37. A displacement of 38 was seen. A mild radiolucent fracture line was seen on the right side of the parasymphysis region and the body of the mandible in relation to 48. Mild displacement of the left condyle was seen with the help of an OPG. The final diagnosis was made of a right parasymphysis fracture and a left body of mandible fracture.

The treatment plan entailed the refracture of the malunited fracture followed by open reduction internal fixation (ORIF), and pre-operatively, intermaxillary fixation (IMF) was initiated. After two days, treatment commenced with intermaxillary fixation concurrent with the extraction of a tooth exhibiting a poor prognosis. After the initial treatment, ORIF was carried out under general anesthesia.

Procedure

**Figure 4:**
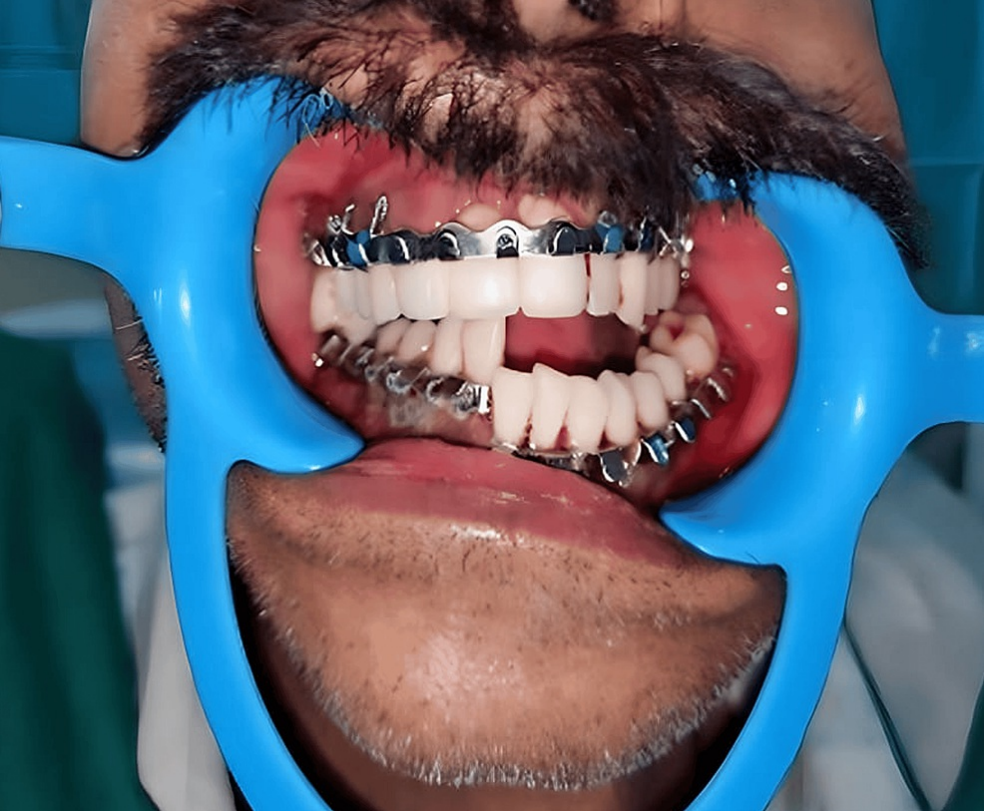
Archbar fixation

Intermaxillary fixation was done using arch bars (Figure 4).

The fracture site was exposed by intraoral vestibular incision and a mucoperiosteal flap was elevated in relation to the fracture region.

**Figure 5:**
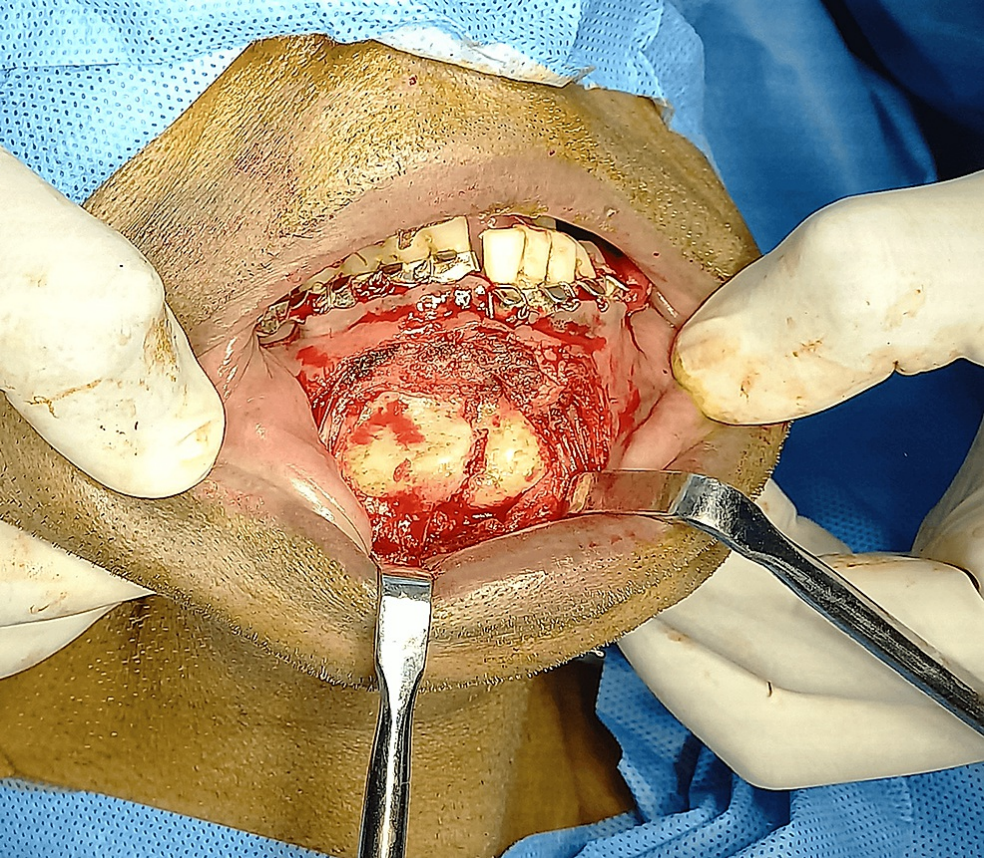
Intraoperative image showing a parasymphysis fracture

**Figure 6:**
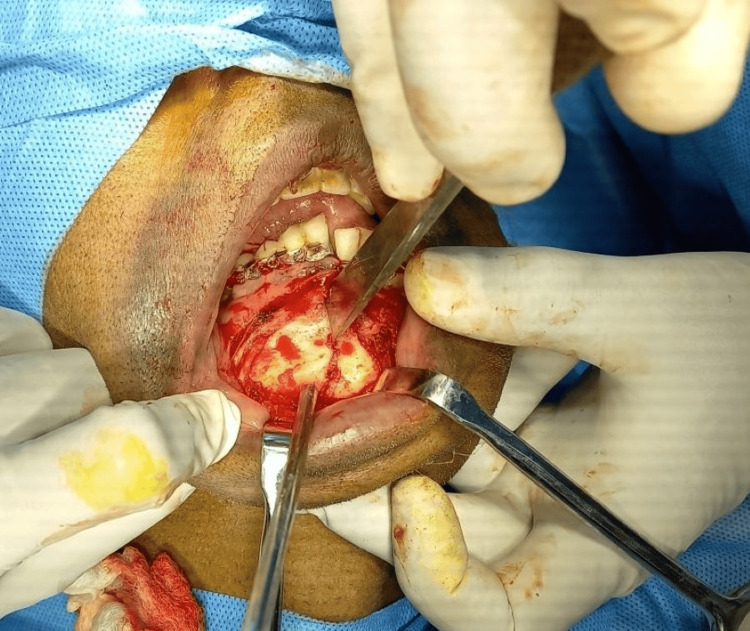
Intraoperative image showing refracturing of the parasymphysis fracture

In the parasymphysis region, refracturing of the malunited bone was done using an osteotome. Smith’s bone spreader was used to separate the fracture segment (Figures 5, 6).

**Figure 7:**
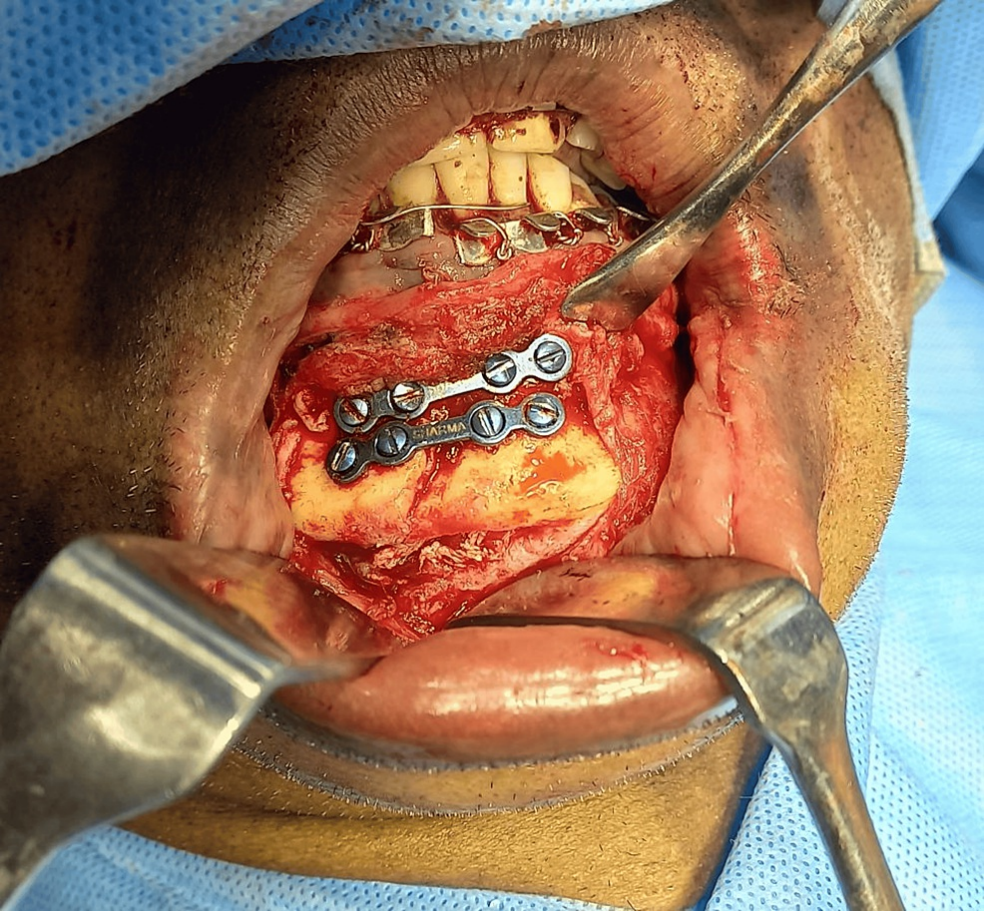
Intraoperative image showing reduction and fixation of the parasymphysis fracture site using two 2 mm titanium plates

The callus was removed from the fractured segment and then the fracture reduction was carried out. The main aim of fracture reduction is to obtain occlusion and cosmesis. Subsequently, the fracture site was approximated, and two 2 mm four-hole titanium plates were secured with 8 mm screws in the approximated fracture segment after achieving occlusion (Figure 7).

**Figure 8:**
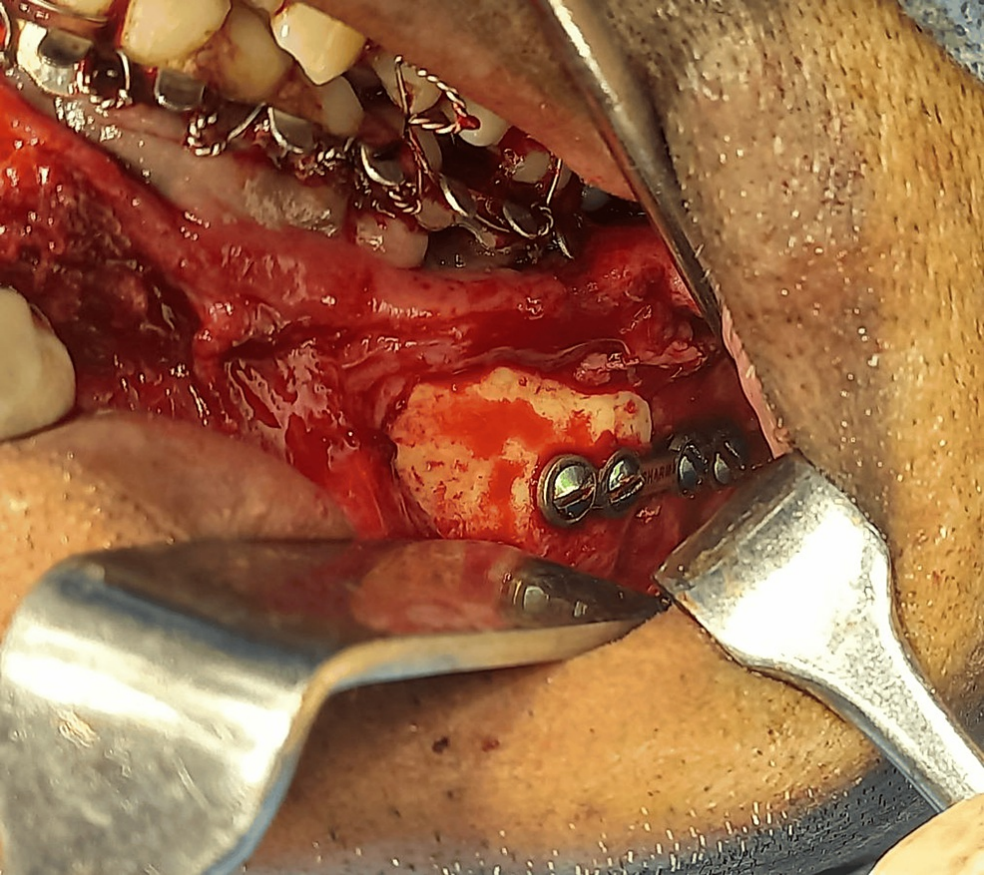
Intraoperative image showing reduction and fixation of the angle of the mandible fracture site using one 2.5 mm titanium plate

In the body of the mandible, the malunited bone was refractured and separated using an osteotome and Smith's bone spreader, respectively. The callus formed was then removed from the fractured segment. Subsequently, the fracture site was approximated, and one 2.5 mm titanium plate was secured with 8 mm screws in the approximated fracture segment after gaining occlusion (Figure 8).

**Figure 9:**
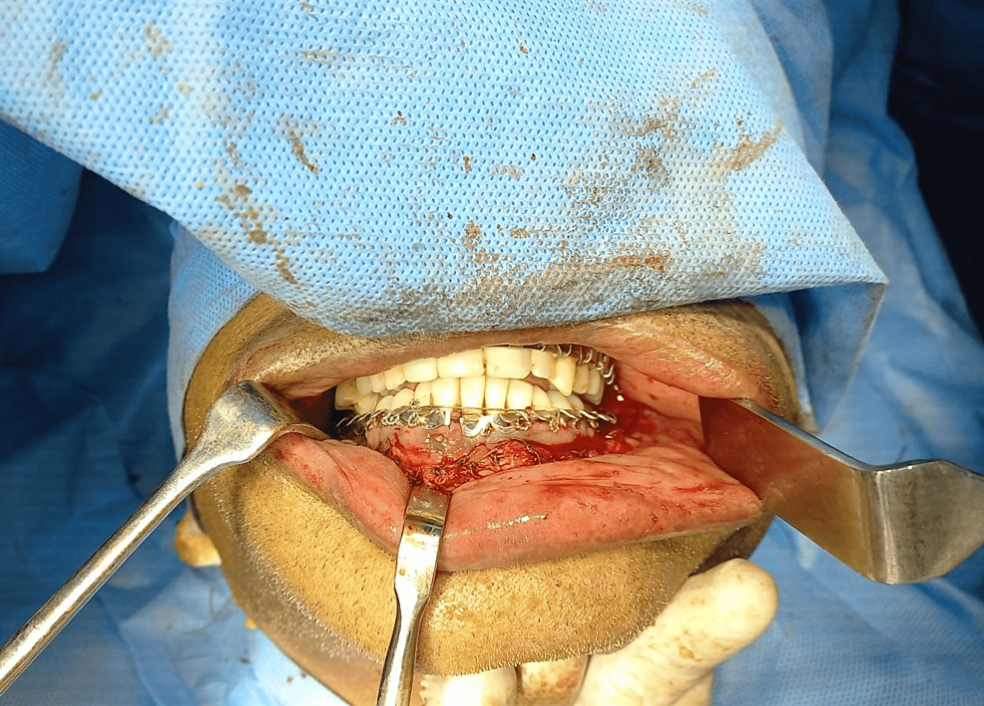
Intraoperative image of post-flap closure with precise occlusion

Then the reflected flap was closed and a simple interrupted suture was placed using 3-0 vicryl (Figure 9).

Saline irrigation was done, and the patient was recalled after two days for a review. During the review, there was no evidence of postoperative swelling, and no gapping was noted between the sutures.

**Figure 10:**
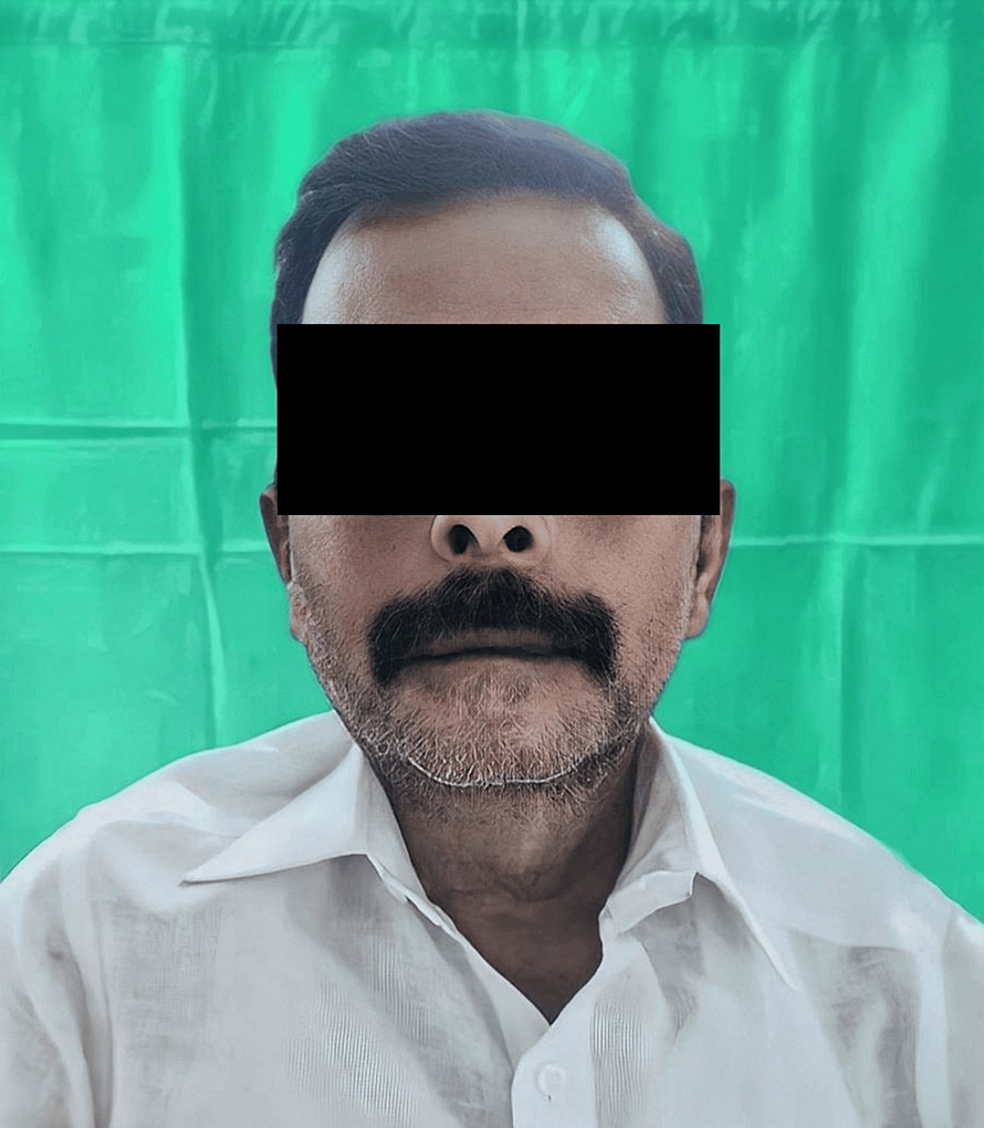
Postoperative image revealing the patient's restored facial symmetry

**Figure 11:**
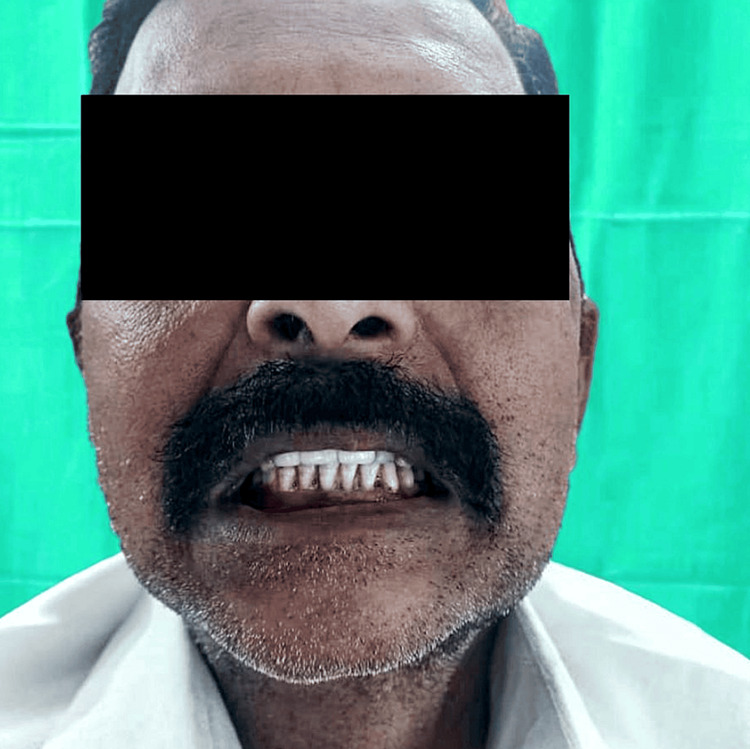
Postoperative image depicting the patient's corrected malocclusion

In the aftermath of the surgical procedure, facial asymmetry was addressed (Figure 10) and the patient achieved a significant improvement in occlusion (Figure 11).

**Figure 12:**
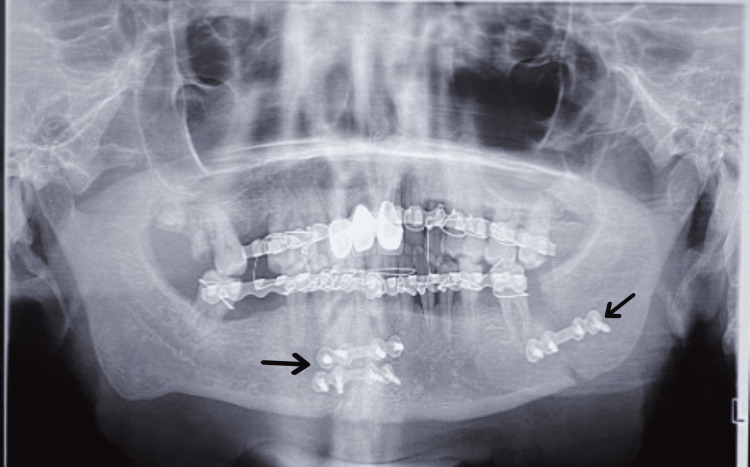
Postoperative OPG revealing successful reduction and fixation of malunited fractures using 2 mm and 2.5 mm titanium plates, highlighted with precision arrows OPG: orthopantomogram

Following the successful procedure, a postoperative OPG was taken (Figure 12), and the patient remained under follow-up for the next three months, during which the healing process was deemed satisfactory. There was an improvement in facial asymmetry, and occlusion was successfully restored.

## Discussion

Malunion of a mandibular fracture can have serious repercussions and should be addressed during primary surgery if at all possible[11]. According to the research, the most successful therapy for post-traumatic malocclusion is an osteotomy on the affected side [12]. Surgical treatment allows for correct morphological and functional reconstruction [13]. Malunion of the fracture might cause occlusion and function to be disrupted. Hence, the fundamental rationale for executing the osteotomy in the therapy of such instances is that it has various advantages, for example, osteotomy at the site increases the probability of restoring the pre-traumatic morphology. There is a somewhat good area of bone contact, allowing the pieces to move freely. Significant recontouring of the bone may be required in order to achieve an optimal form in the end. Bone grafting is not required with this treatment unless the remodeling procedure creates a gap. As a general rule, separations of more than 1.5 cm will not heal without the insertion of a bone graft. Unless there are extenuating circumstances, no attempt should be made to directly appoint the ends of the bones because doing so will considerably alter the occlusion [11].

Due to accompanying systemic problems that necessitate more urgent attention, facial fractures from traumatic traumas frequently necessitate subsequent treatment [14]. Dingman and Natvig addressed the use of various osteotomies in conjunction with the IMF to restore form and function in cases of residual deformity following malunion [15]. The timing of intervention and the status of the soft tissue envelope are crucial principles for special discussion in every case of PTRD [16]. Mandibular fractures were thought to need treatment within 24 to 48 hours, according to conventional wisdom [17]. Delaying therapy may raise the risk of technical difficulties, infection, malocclusion, and malunion [18]. This involves creating a wide exposure to visualize bony irregularities, making precise incisions, and reconnecting soft tissues. Bony deformities can be corrected via osteotomy, onlay grafting, or a combination of the two methods. Osteotomy is frequently used when an individual component of the face skeleton has normal morphology but is in an aberrant position (displacement). With osteotomy, the mandibular position can be restored [19].

In our situation, osteotomy and mini plates were employed to treat a malunited parasymphysis fracture and a body of mandible fracture. The treatment resulted in maximum bone contact. Consequently, reconstruction plates are not implicated in the malunited fracture in this case. Due to several limitations associated with the harvesting process, neither bone nor alloplastic transplant was used. Common consequences, such as donor-site pain, increased blood loss, extended operative duration, and the potential for donor-site infection, were considered.

## Conclusions

Conservative treatment cannot provide anatomically correct posture in adults after a fracture. The larger the deviation of the mandible, the bigger the morphological deviations. As a result, open reduction with fixation is indicated as the therapy of choice in adult patients with mandibular fractures. Severe dislocation or luxation of the mandible, functional impairment, and malocclusion are all indications for surgical therapy. To summarize, mandibular malposition can be corrected following malunited fractures by executing one of several types of conventional osteotomies. We were able to achieve normal occlusion, correct mandibular position, and facial morphology harmonization in our cases by placing 2 mm plates in the parasymphysis region and 2.5 mm plates in the mandibular angle without using bone or alloplastic grafts.
